# Insulin Stimulated-Glucose Transporter Glut 4 Is Expressed in the Retina

**DOI:** 10.1371/journal.pone.0052959

**Published:** 2012-12-21

**Authors:** Gustavo Sánchez-Chávez, Ma. Teresa Peña-Rangel, Juan R. Riesgo-Escovar, Alejandro Martínez-Martínez, Rocío Salceda

**Affiliations:** 1 División Neurociencias, Instituto de Fisiología Celular, Universidad Nacional Autónoma de México, México, Distrito Federal, México; 2 Departmento de Neurobiología del Desarrollo y Neurofisiología, Instituto de Neurobiología, Universidad Nacional Autónoma de México, Querétaro, México; 3 Universidad Autónoma de Ciudad Juárez, Ciudad Juárez, Chihuahua, México; Max-Delbrück Center for Molecular Medicine (MDC), Germany

## Abstract

The vertebrate retina is a very metabolically active tissue whose energy demands are normally met through the uptake of glucose and oxygen. Glucose metabolism in this tissue relies upon adequate glucose delivery from the systemic circulation. Therefore, glucose transport depends on the expression of glucose transporters. Here, we show retinal expression of the Glut 4 glucose transporter in frog and rat retinas. Immunohistochemistry and *in situ* hybridization studies showed Glut 4 expression in the three nuclear layers of the retina: the photoreceptor, inner nuclear and ganglionar cell layers. In the rat retina immunoprecipitation and Western blot analysis revealed a protein with an apparent molecular mass of 45 kDa. ^14^C-glucose accumulation by isolated rat retinas was significantly enhanced by physiological concentrations of insulin, an effect blocked by inhibitors of phosphatidyl-inositol 3-kinase (PI3K), a key enzyme in the insulin-signaling pathway in other tissues. Also, we observed an increase in ^3^H-cytochalasin binding sites in the presence of insulin, suggesting an increase in transporter recruitment at the cell surface. Besides, insulin induced phosphorylation of Akt, an effect also blocked by PI3K inhibition. Expression of Glut 4 was not modified in retinas of a type 1 diabetic rat model. To our knowledge, our results provide the first evidence of Glut4 expression in the retina, suggesting it as an insulin- responsive tissue.

## Introduction

The mammalian retina is characterized by high-energy requirements, relaying mainly on glucose as the principal energy source to meet demand [1], yet the mechanisms regulating glucose homeostasis within the retina remain largely unknown. Glucose transport should undoubtedly play a principal role. Alterations in glucose supply could, therefore, potentially change retinal energy metabolism and result in complications. Indeed, retinopathies are one clinical manifestation of long standing diabetes mellitus [2].

Glucose transport in eukaryotic cells occurs mainly through facilitated diffusion glucose transporters (Glut proteins). To date, thirteen Glut isoforms have been identified and cloned, with distinct physiological features and tissue distribution [3]. In the retina, Glut 1 has been found in endothelial, retinal pigment epithelium (RPE) and photoreceptor cells [4], [5], [6], [7]. Glut 2 is expressed at the apical ends of Müller cells [8], and Glut 3 in the inner synaptic layer of the human [9] and the rat retinas (Salceda, unpublished).

A major action of insulin is to promote glucose metabolism, an effect largely due to increased glucose transport. The insulin-regulated glucose transporter Glut 4 is expressed mainly in insulin-responsive tissues, i.e., adipose and muscle tissues [10], where it mediates glucose uptake in response to insulin stimulation. However, Glut 4 has also been reported in neurons [11], [12]. In the retina, insulin and its receptor [13], [14] have been reported, yet their function is not understood. Therefore, we carried out different experimental approaches including immunohistochemical and *in situ* hybridization to characterize Glut 4 expression in the retina.

## Materials and Methods

### Experimental Animals

Adult Long Evans rats (170–200 g) and frogs (*Rana pipiens*) were used. Animals were maintained under standard laboratory conditions (21°C±1, 12 h light-dark cycle) with food and water *ad libitum*. All experiments were conducted between 9∶30 and 12∶00 pm.

All procedures were conducted in accordance with the Mexican Institutes of Health Research rules (DOF. NOM-062-Z00-1999), the National Institutes of Health Guide for the Care and Use of Laboratory Animals (NIH publication No. 80–23, revised 1996), as well as the Association for Research in Vision and Ophthalmology Statement on the Use of Animals in Ophthalmic and Vision Research. The Ethics Committee for Animal Experiments of our Institution approved the experimental protocol. All efforts were made to minimize animal suffering, and to reduce the number of rats used.

### Diabetes induction

Diabetes was induced in adult Long Evans rats by a single intraperitoneal administration of streptozotocin (65 mg/ Kg) in citrate buffer, pH 4.5, as described [15]. Animals were used 20 and 45 days after streptozotocin treatment when the existence of diabetes was inferred from impaired growth and polyuria [16]. Animals were decapitated, eyes excised and the anterior part was removed; retinas were peeled away gently using fine forceps.

### Western Blotting

Rat retinas were homogenized in RIPA lysis buffer (10 mM Tris-HCl pH 7.5, 158 mM NaCl, 1 mM EGTA, 10 mM Na_2_MoO_4_, 25 mM NaF, 1 mM Na_3_VO_4_, 1 mM phenylmethylsulfonyl fluoride [PMSF], 1 mM EDTA, 1 μg/ml leupeptin, 1 μg/ml aprotinin, 2% Triton X-100, 0.2% SDS). The homogenates were centrifuged 20 min at 17,000 g and pellets discarded. Approximately 100 μg of protein sample was subjected to 10% SDS-PAGE and transferred to PVDF Immobilon membranes (Millipore Corp.) [17]. The amount of protein loaded was monitored by 1% Ponceau Red staining. Molecular weight markers were identified and subsequently the membranes washed in TBS (20 mM Tris-HCl, 136 mM NaCl, pH 7.6) to remove the Ponceau Red stain. To block, membranes were treated with TBS-T (TBS containing 0.1% Tween 20 and 5% nonfat milk) for 2 h; they were then incubated overnight at 4°C with a mouse-monoclonal antibody against Glut 4 (1∶500, Bio-Trend; or 1∶100, Cell Signaling) in TBS-T containing 0.25% BSA. After 3 washes in TBS-T and 1% nonfat milk, the membranes were incubated for 2 h at room temperature with horseradish peroxidase-conjugated secondary anti-mouse (1∶40000, GE Healthcare Ltd) in TBS-T containing 0.25% BSA and 1% nonfat milk. Antibody staining was detected using the Chemiluminescent HRP Substrate (Immobilon Western Chemiluminescent HRP Substrate, Millipore Corp.) according to the manufacturer's instructions. The intensity of signals revealed on Hyperfilm ECL (GE Healthcare Ltd.) was digitized with an Alpha DigiDoc RT (Alpha Innotech.) and analyzed using relative optical densities derived from a computer-assisted densitometry program (Alpha Ease FC Stand Alone; Alpha Innotech.). Protein loading was normalized to actin using a monoclonal primary antibody (1∶500; Millipore-Corp.) and a secondary antibody (1∶8000 GE Healthcare Ltd.).

We also performed immunoprecipitation studies using rabbit polyclonal anti Glut 4 (Santa Cruz Biotech.) coupled to sepharose beads-protein A [18]. The protein A-anti Glut 4 complex was denatured in Laemmli's sample buffer and resolved through 10% polyacrilamide gels electroblotted to PVDF membranes. Afterwards, membranes were incubated with a mouse monoclonal anti-Glut 4 antibody (1∶500, Bio-Trend). Further steps were done as described above. For each SDS-PAGE, proteins from muscle extracted from normal rats as described above were included as positive controls.

### Immunohistochemistry

Eyes were rapidly removed and eye cups fixed by immersion in 4% paraformaldehyde in 100 mM phosphate-buffered saline (PBS), pH 7.4. After 2 h, tissue was rinsed in PBS and cryoprotected overnight at 4°C in 30% sucrose-PBS. Ten µm cryostat sections were incubated for 45 min with PBS containing 5% bovine serum albumin and 0.1% Triton X-100. Sections were then incubated overnight at 4°C with a rabbit polyclonal antibody against Glut 4 (diluted 1∶ 200, Santa Cruz Biotechnology, Inc.). This was followed by a 2 h incubation with a secondary TRITC-conjugated goat anti rabbit (1∶150, Sigma, Co.). In controls, primary antibody incubation was omitted. Sections were mounted in glycerol and examined using a Nikon microscope (Nikon Corp., Tokyo, Japan) and photographed with a Nikon DXM1200 digital camera (Nikon Corp., Tokyo, Japan).

### Synthesis of riboprobes

To synthesize riboprobes, we used a Glut 4 partial cDNA (#UI-R-BJ2-bqh-d-02-0-UI.s1) purchased from the University of Iowa Program for Rat Gene Discovery and Mapping. This cDNA maps to the 3′ end of full-length Glut 4 cDNA (full-length is 2506 bp long). UI-R-BJ2-bqh-d-02-0-UI.s1 is only 505 bp long, and BLAST searches against the rat genome and rat reference cDNAs yielded a single match, with the Glut 4 locus/cDNA. Riboprobes were synthesized using the DIG RNA labeling kit (Roche Diagnostics) according to the manufacturer's instructions. We used 1 μg of gel-purified linearized Glut 4 cDNA cloned in the pT7T3D-Pac plasmid. For synthesis, the plasmid was digested with Not I for the sense probe and EcoRI for the antisense probe. After synthesis, riboprobes were precipitated with 100 mM LiCl in ethanol, washed with 70% ethanol and resuspended in hybridization buffer, and stored at −20°C until use. Dot-blots were carried out to estimate extent of labeling.

### *In situ* hybridization

*In situ* hybridizations were carried out with digoxigenin labelled riboprobes and frozen tissue sections according to the manufacturer's instructions (Roche Diagnostics), as described previously [19]. Briefly, tissue sections obtained from fixed, cryostat-sectioned retinas were dried at 60°C for 15 minutes, then post-fixed with 4% paraformaldehyde in PBS for a further 15 minutes. Sections were then washed with PBT (PBS-Tween 20 at 0.1%), then with a 1∶1 mixture of PBT hybridization solution and then incubated for 1 h with hybridization solution at 55°C. Hybridization solution is 50% formamide, 5×SSC, 100 microgram/ml. salmon sperm DNA and 0.1% Tween 20. Hybridization was then carried overnight at 55°C with heat-denatured riboprobes in hybridization solution. Sections were then washed for 1 h at 60°C in fresh hybridization solution, then washed several times at 60°C in PBT, blocked with PBS with 5% fetal calf serum at room temperature for 10 minutes, and incubated with anti-dig antibody 1∶2000 in the same PBS-5% fetal calf serum solution for 2 h at room temperature. Sections were then washed with PBT, and then with detection solution. Detection solution is 100 mM NaCl, 50 mM MgCl_2_ and 100 mM Tris-HCl, pH 9.5. Sections were then incubated with BCIP-NBT reagent in detection solution in the dark at room temperature, and reaction monitored. Sections were then mounted in PolyMount (PolySciences, Inc.). Antisense probes were used in parallel with their respective sense controls. We analyzed at least 10 sections per retina of each of eight animals, done in three separate experiments. As a further control, cerebellar sections were hybridized in parallel with the same Glut 4 riboprobes, and results corroborated earlier reports of Glut 4 expression in this tissue [11], [12].

### Glucose uptake

Isolated rat retinas were incubated for 20 min at 37°C in 1 ml Krebs Ringer-bicarbonate (118 mM NaCl, 1.2 mM KH_2_ PO_4_, 4.7 mM KCl, 2.5 mM CaCl_2_, 1.17 mM MgSO_4_, 25 mM NaHCO_3_, 5.6 mM D-glucose, pH 7.4) containing 0.25 μCi of D-[^14^C (U)]-glucose ( 265 mCi/mmol, New England Nuclear, Boston, MA). At the end of the incubation, the tissue was rinsed with cold medium, weighed and dissolved in 0.5 ml of 1% sodium dodecyl sulfate. Radioactivity in the solubilized tissue was measured in a liquid scintillation spectrometer. Volume of extracellular space was estimated as described [15], and subtracted from the uptake values.

### Cytochalasin B binding

^3^H-Cytochalasin B binding was performed in whole retinas by a modification of the method described by Cushman [20]. Intact retinas were incubated at 37°C in a Krebs Ringer containing 300 nM ^3^H-cytochalasin B (New England Nuclear, Boston, MA). Cytochalasin E (2 μM, Aldrich Chemical Co.) was added to all conditions to saturate all other binding sites of cytochalasin B not related to glucose transporters. Non-specific ^3^H-cytochalasin B binding was determined by incubation in the absence or presence of 5 mM D-glucose. After 20 min incubation, the tissue was rinsed with cold medium, weighed and dissolved as described above. Radioactivity in the solubilized tissue was determined by liquid scintillation counting.

### Akt-phosphorylation

Retinas were incubated for different periods at 37°C in 1 ml Krebs Ringer in the absence or presence of insulin (10 ng/ml), and also in the absence or presence of 10 μM of LY294002. At the end of the incubation, tissues were homogenized in lysis RIPA buffer and processed for Western blot as described above. A rabbit anti-Akt (1∶4000, Cell Signaling), and rabbit anti phospho-Akt (Ser473) (p-Akt, 1∶500, Cell Signaling) were used with an anti rabbit horseradish peroxidase-conjugated (1∶10000, GE Healthcare Ltd.) secondary antibody.

### Protein content and glucose assessment

Protein content was determined by the method of Lowry et al. [21] using a commercial assay kit (BioRad, Hercules, CA) with bovine serum albumin as standard. Blood glucose concentration was determined with a blood glucose monitor (Accu-chek, Roche).

### Statistical Method

Biochemical data are presented as mean ± SEM of at least five separate experiments. Significance was determined by either t-test or one-way ANOVA analysis, followed by Tukey's post hoc test.

## Results

**Figure 1:**
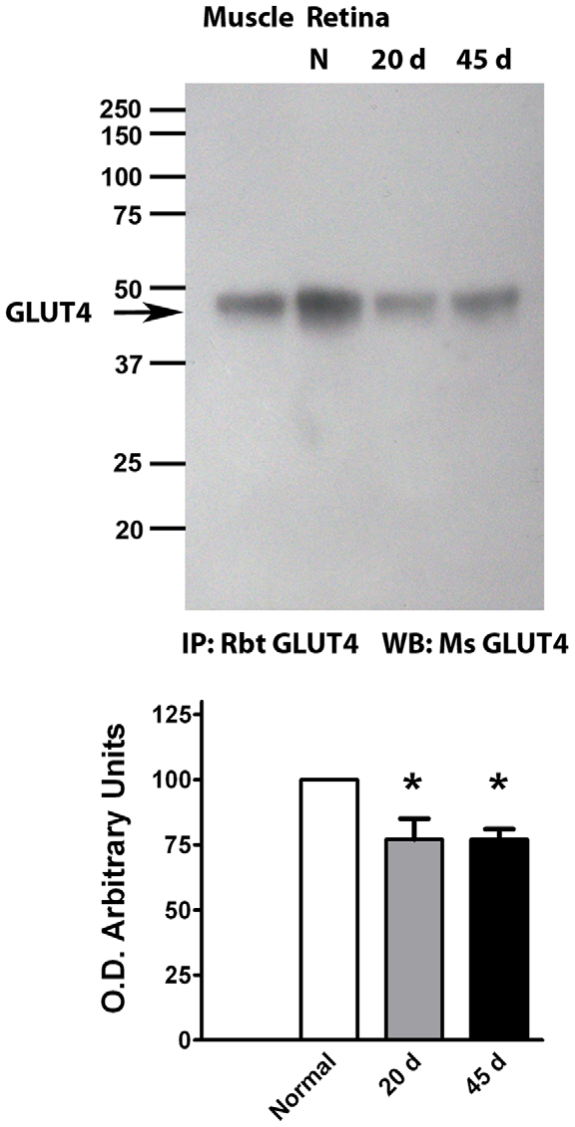
Glut 4 expression in the retina. Immunoprecipitation (IP) followed by Western blotting (WB) and detection. 100 μg protein were loaded. Quantification of data is presented as the mean ± SEM of at least six animals per group. M, muscle; N, normal; 20d, 20 days diabetic rats; 45d, 45 days diabetic rats.

We immunoprecipitated and blotted Glut 4 form muscle and retinal homogenates using a polyclonal anti Glut 4 antibody (Fig. 1). We also performed Western blots with two different anti Glut 4 monoclonal antibodies. Both types of antibodies revealed an immunoreactive band of similar molecular mass (45 kDa), yet the monoclonal antibodies revealed additional bands, and were not used further (Fig. S1). The 45 kDa Glut 4 molecular mass band is close in size to the one reported for skeletal muscle [22] (Fig. 1). We observed statistically significant slight decreases (25%) in Glut4 expression in streptozotocin-diabetic rat retinas (Fig. 1).

**Figure 2:**
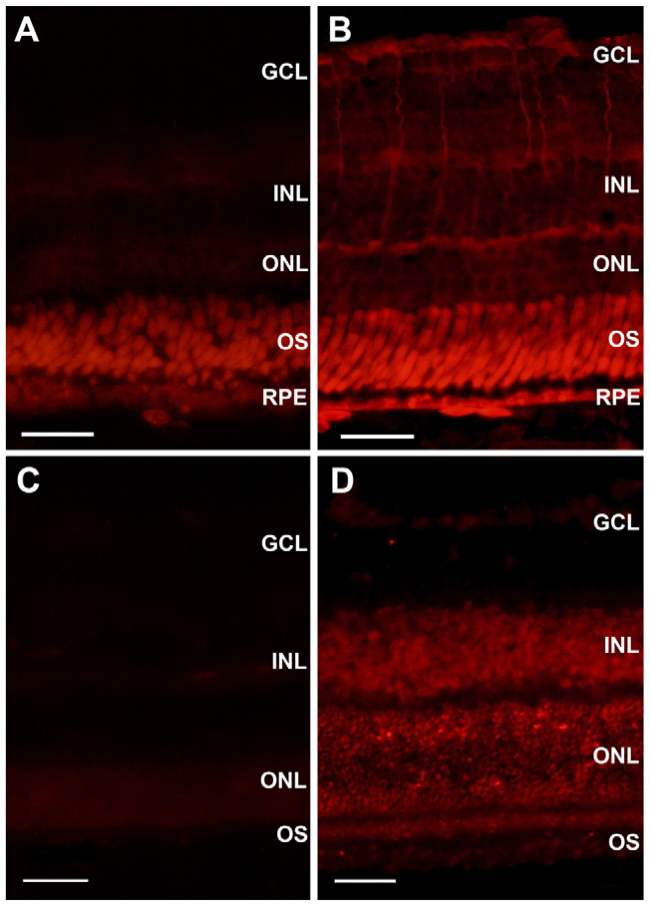
Immunodetection of Glut 4 in the retina. Representative photomicrographs of vertical sections of frog (A, B) and rat (C, D) retinas, showing immunoreactivity to anti Glut 4 antibody (B and D, respectively). A, C are negative controls, in which primary antibody was omitted. At least five sections of five animals were analyzed. GCL, ganglionar cell layer; INL, inner nuclear layer; ONL, outer nuclear layer; OS, outer segments; RPE, retinal pigment epithelium. Bar represents 50 μm.

We then studied cellular distribution of this transporter in rat and frog retinas. Immunolabeling of rat retinal sections with anti Glut 4 antibody revealed a strong signal in the inner nuclear layer (INL), the outer nuclear layer (ONL), and with lower intensity, in the ganglion cell layer (GCL) (Fig. 2). In the frog retina, Glut 4 labeling was observed in the photoreceptor layer, glial Müller cells fibers, and in the GCL (Fig. 2). Interestingly, we also observed some labeling in the frog RPE.

**Figure 3:**
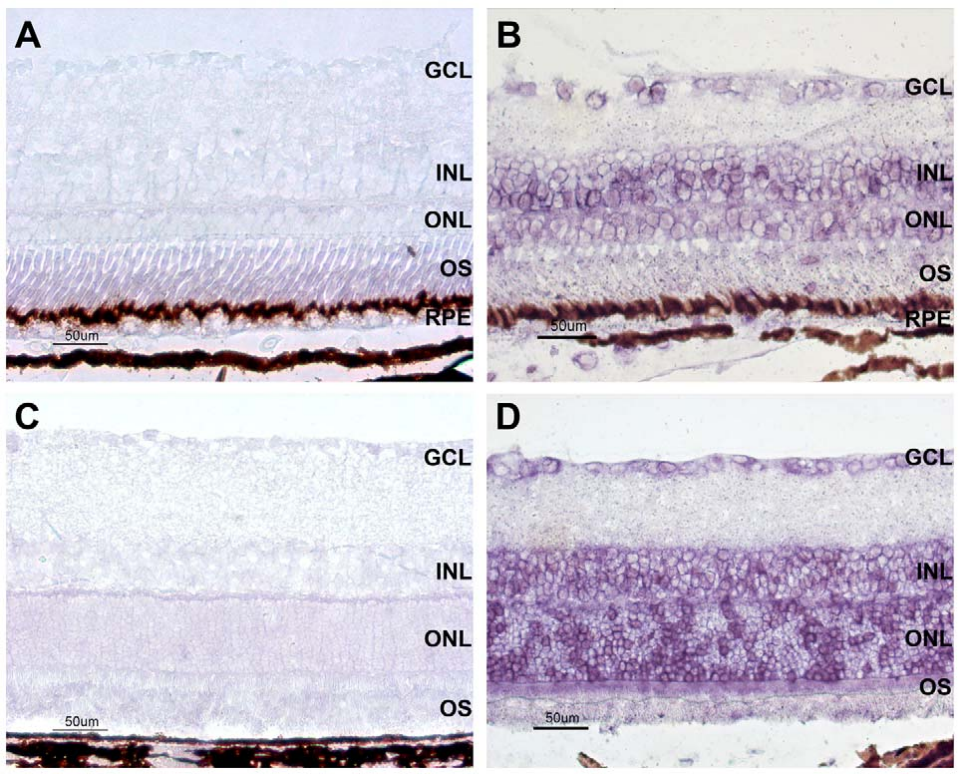
Glut 4 mRNA expression in the retina. Representative sections of frog (A,B) and rat (C,D) retinas processed for in situ hybridization of Glut 4 mRNA. Low magnification photographs of alkaline phosphatase developed retinal sections showing distribution of Glut 4 mRNA (B and D, respectively). Sense probe (controls) are A and C, whereas B and D were hybridized with antisense probes. GCL, ganglionar cell layer; INL, inner nuclear layer; ONL, outer nuclear layer; OS, outer segments; RPE, retinal pigment epithelium. Bar represents 50 μm.

We then studied expression of Glut 4 mRNA in rat and frog retinas. *In-situ* hybridization with Glut 4 mRNA antisense probes in rat and frog retinas, consistent with antibody results, showed a positive signal in the GCL, INL and ONL. Labeling was irregular, however; several cells presented high levels of transcript expression, while others did not (Fig. 3).

**Figure 4:**
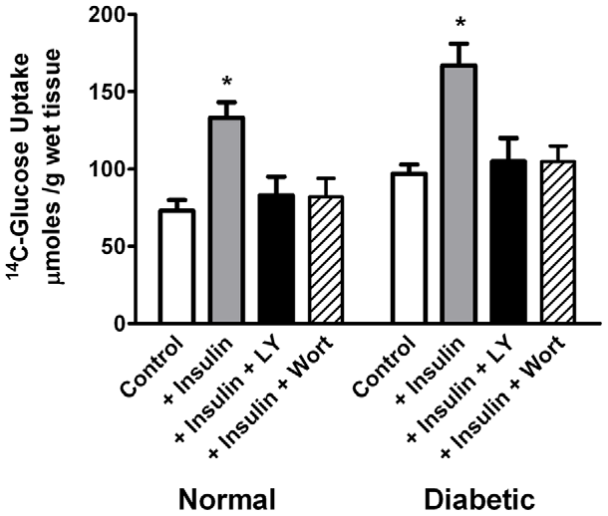
Characterization of ^14^C-Glucose uptake by retinas of normal and streptozotocin-induced diabetic rats. Tissues were incubated for 20 min in a Krebs medium with or without insulin (10 ng/ml). The effects of LY294002 (LY, 10 μM) and wortmannin (Wort, 50 nM) on incubations with added insulin are shown. Each value is the mean ± SEM of five animals. * Significantly different from control at p<0.02.

It is known that insulin induces glucose transport by increasing Glut 4 recruitment to the plasma membrane [23]. Therefore, we studied the effect of insulin on retinal glucose uptake. Isolated rat retinas incubated for up to 20 min in a normal Krebs medium containing ^14^C- glucose accumulated 70±7 nmol/mg prot. Addition of physiological insulin concentrations (10 ng/ml) to the incubation medium significantly enhanced ^14^C-glucose uptake (Fig. 4). The phosphatidyl-inositol 3-kinase (PI3K) inhibitors, LY294002 (10 μM) or wortmannin (50 nM), prevented this insulin-mediated ^14^C-glucose uptake increase. Diabetic rat retinas behaved similar to normal animals (Fig. 4).

The extent of ^3^H-cytochalasin B binding allows determination of the proportion of membrane surface glucose transporters. We characterized ^3^H-cytochalasin B binding in intact rat retina. In three different experiments we found that rat retina incubated in Krebs medium bound 103±6.6 pmol/g wet tissue of ^3^H- cytochalasin B. Addition of insulin (10 ng/ml) to the incubation medium stimulated binding of ^3^H-cytochalasin B by 30% (142±5.5 pmol/g wet tissue, p<0.002).

**Figure 5:**
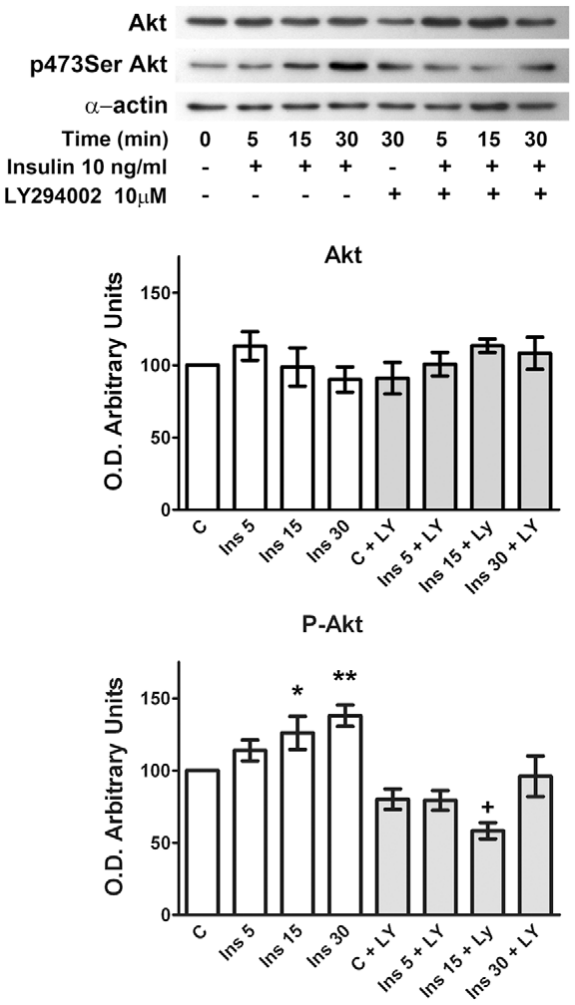
Time course of insulin-induced phosphorylation of Akt. The figure shows a Western blot of a representative experiment (top) and densitometric analyses for total (Akt) and phosphorylated-Akt (p-Akt). Retinas were incubated in Krebs medium in the absence or presence of 10 ng/ml insulin, and in the presence of LY294002 (LY, 10 μM). Data are the mean ± SEM of five different experiments, each one carried out in duplicate. * p<0.03; ** p<0.0001; + p = 0.0001; all with respect to the control (C).

We then studied the effect of insulin on Akt phosphorylation. Incubation of rat retinas in a Krebs medium containing insulin (10 ng/ml) induced time-dependent Akt phosphorylation. This insulin effect was inhibited by the PI3K inhibitor LY294002 (10 μM) (Fig. 5).

## Discussion

Glucose is the major fuel driving energy metabolism in vertebrate retinas [1]. This tissue has the capacity to transport exogenous glucose from circulation, since the Glut 1 glucose transporter is widely distributed in the retina and RPE [5], [6], [7]; in addition, Glut 2 and Glut 3 are also present [8], [9].

Here, we show expression of Glut 4 in rat and frog retinas by Western blot, immunohistochemistry, and *in situ* hybridization. We found expression of Glut 4 in all cell layers of frog and rat retinas. Of particular interest is the presence of Glut 4 in photoreceptor cells. Retinal insulin receptor possesses tyrosine kinase activity in purified bovine rod outer segments [13]. Rajala and coworkers found an interaction between the insulin receptor and PI3K in bovine rod outer segments [24], an interaction stimulated by insulin in vitro [25]. In addition, insulin can stimulate taurine uptake [26], the main free amino acid in the retina, essential for maintenance of the structural and functional integrity of this tissue [27].

We did not find expression of Glut 4 in retinal vessels. These results are consistent with those where lack of Glut 4 expression in endothelial cells was previously noted [4], [28].

Glut 4 expression in the frog retina suggests that insulin or other insulin-related peptides may have an evolutionary conserved role in retinal physiology. Indeed, a variety of studies suggest that insulin plays a major role in development as a trophic factor [29], [30], [31]. Moreover, insulin receptor substrate-1, the major substrate of the insulin receptor, is necessary for amphibian eye development [32].

Activation of the insulin receptor leads to phosphorylation of the insulin receptor substrates, creating sites for PI3K binding. This, in turn, activates PKB/Akt, resulting in an increase in Glut 4 transporters at the plasma membrane [33]. Therefore, we also studied the effect of insulin in retinal glucose uptake. We demonstrate insulin-dependent increased glucose uptake in the isolated rat retina, an effect blocked by PI3K inhibitors [34]. ^3^H-cytochalasin B binding increased 30% in rat retinas incubated with insulin, strongly suggesting insulin-induced recruitment of the glucose transporter to the plasma membrane from an internal pool, as reported in muscle fibres and adipocytes [20], [23]. Although generally it may be straightforward to measure directly membrane bound cytochalasin B in plasma membranes, it is a very difficult experiment to perform in the retina. To further substantiate our contention that insulin redirects Glut 4 transporters in the retina, we also show insulin induced PI3K-dependent Akt phosphorylation that would then result in Glut 4 transporter movement to the cell surface membrane. Altogether, these results suggest that the insulin induced increase in glucose uptake and the cytochalasin B binding observed are related to insulin signaling. This implies that the retina is an insulin-sensitive tissue.

The retinal response to insulin observed here is nonetheless limited compared to other tissues. Our results indicate that insulin increased the basal glucose transport capacity of the retina by 30%. Assuming that this increase is due to Glut 4 recruitment to the plasma membrane, this apparent small response can be explained by a lower total number of transporters or to a different distribution between the internal pool and the one at the membrane surface in these cells. Alternatively, it can be due to differential insulin response of distinct retinal cell types expressing the transporter. Insulin could be secreted from retinal cells, and employed in autocrine responses, since insulin and its receptor have been reported both in blood vessels [35] and the neural retina [13], [14].

Interestingly, although Glut 4 expression decreased slightly in the diabetic rat retina, insulin-stimulated glucose uptake was not modified by diabetic conditions. These results could be due to differences in glucose transporter expression in retinal cells and extent of affectation of retinal cells in the diabetic state. These results agree with previous ones where kinetic properties of deoxy-D-glucose uptake were similar in normal and diabetic rat retinas [15]. Likewise, expression of Glut 4 was not modified in muscle from streptozotocin-induced diabetic rats [36] or type 1 diabetic patients [37], compared with controls.

As is true in the brain [11], [12], existence of multiple glucose transporter isoforms in the retina with different kinetic properties and expression patterns at the cell surface suggest complex regulation of glucose metabolism, providing a basis for fine tuning glucose uptake metabolism and signal generation. Since retinal function is highly dependent on circulating glucose levels, glucose transport isoforms may play specific roles in glucose handling, metabolism, gene expression or differentiation, each one likely to operate through different regulatory avenues. Increases in neural activity are associated with increases in brain glucose uptake [38], [39], and Glut 4 is postulated to facilitate neuronal function during increased metabolic demand [40]. Similar to muscle and adipose tissue, insulin-mediated Glut4 translocation has been demonstrated in the brain [41], [42], [43]. In addition, Glut 4-mediated glucose sensing is postulated to be important under hypoglycemic conditions [44]. Glut 4 might function in the retina to deliver glucose to meet energy expenditures of particular retinal processes associated with the maintenance of glucose supply under hypoglycemic conditions, despite the abundance of retinal Glut 1, since the affinity of Glut 1 for glucose is lower than that of Glut 4. In this regard, recent evidence shows that under hypoglycemic conditions retinal energy supply may be compromised [45], [46], [47]. Expression of Glut 4 in the retina thus opens new roles for insulin in retinal physiology.

## Supporting Information

Figure S1 **Western blots of muscle and retinal homogenates (100 μg protein) using two different anti Glut 4 monoclonal antibodies (1∶100, Cell Signaling, left and 1∶500, Bio-Trend right).** Besides the 45 kDa band, additional bands are seen. Anti actin antibodies were used to re-probe the same membranes to assess loading amounts. Quantification of data is presented as the mean ± SEM of normalized data from six different experiments with at least six animals per group. M, muscle; N, normal; 20d, 20 days diabetic rats; 45d, 45 days diabetic rats. * p<0.0001.
(TIF)
