# Supplementary figures and images for: Insulin Stimulated-Glucose Transporter Glut 4 Is Expressed in the Retina
Source: PLoS One. 2012 Dec 21;7(12):e52959. doi: 10.1371/journal.pone.0052959 (PMC3528717; doi:10.1371/journal.pone.0052959)

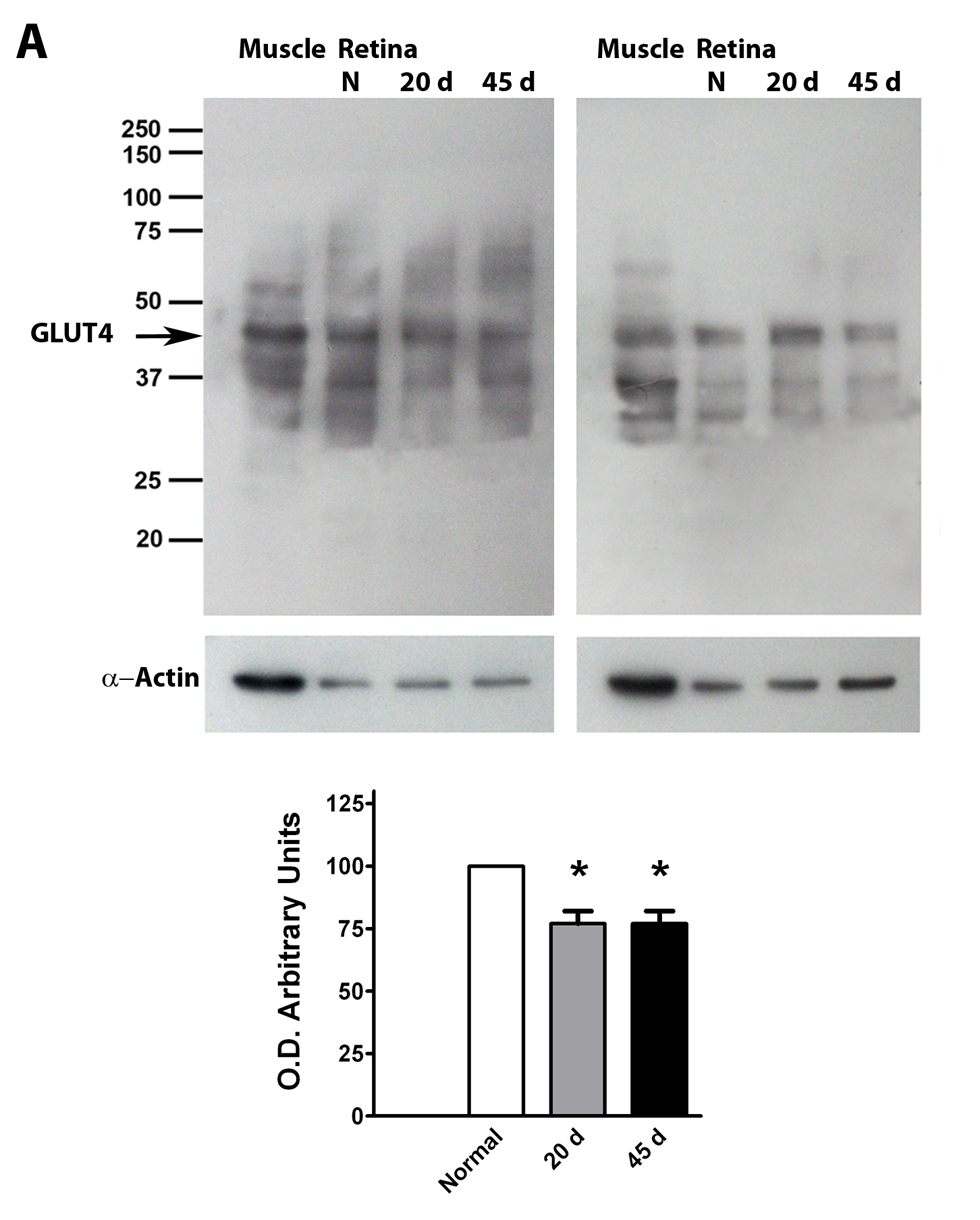

Supplement: Figure S1 — Western blots of muscle and retinal homogenates (100 μg protein) using two different anti Glut 4 monoclonal antibodies (1∶100, Cell Signaling, left and 1∶500, Bio-Trend right). Besides the 45 kDa band, additional bands are seen. Anti actin antibodies were used to re-probe the same membranes to assess loading amounts. Quantification of data is presented as the mean ± SEM of normalized data from six different experiments with at least six animals per group. M, muscle; N, normal; 20d, 20 days diabetic rats; 45d, 45 days diabetic rats. * p<0.0001. (TIF) [file pone.0052959.s001.tif]
